# A limited sampling strategy for estimating busulfan exposure in pediatric hematopoietic stem cell transplantation

**DOI:** 10.3389/fphar.2025.1540139

**Published:** 2025-02-17

**Authors:** Chenhong Jia, Yabin Qin, Yu Han, Weijing Ding, Yuntao Pei, Yile Zhao

**Affiliations:** Department of Pharmacy, Hebei Children’s Hospital, Shijiazhuang, Hebei, China

**Keywords:** busulfan, pediatric hematopoietic stem cell transplantation, limited sampling strategy, therapeutic drug monitoring, area under the concentration-time curve

## Abstract

**Background:**

Busulfan (Bu) is the foundation of conditioning regimens for pediatric hematopoietic stem cell transplantation (HSCT). Evidence indicates that the efficacy and side effects of Bu are intimately tied to the area under its concentration-time curve (AUC). Given its cytotoxic nature and a small therapeutic index, coupled with marked inter-individual pharmacokinetic variability, Bu requires therapeutic drug monitoring to facilitate individualized therapy. However, research investigating the relationship between Bu exposure and clinical outcomes among the Chinese population remains scarce. This study aimed to develop a limited sampling strategy (LSS) for estimating Bu exposure in pediatric HSCT recipients using multiple linear regression (MLR) analysis to predict the AUC_0-360_.

**Methods:**

We enrolled 26 pediatric patients who underwent Bu-based conditioning for HSCT. Blood samples were collected at 11 time points after Bu infusion. Pharmacokinetic parameters were calculated using non-compartmental methods. MLR models were developed using 1–4 sampling points to predict the AUC_0-360_. Model accuracy was assessed using the Jackknife and Bootstrap methods, with consistency evaluated via intraclass correlation coefficient (ICC) and Bland–Altman (BA) analyses.

**Results:**

The mean ± standard deviation (SD) for AUC_0-t_, mean residence time _0-t_, clearance, and volume of distribution were 845.54 ± 111.03 μmol min/L, 181.37 ± 10.55 min, 0.23 ± 0.04 L/h/kg, and 0.73 ± 0.15 L/kg, respectively. Models with 2–4 sampling points showed improved prediction accuracy compared to single-point models. The four-point model (60, 135, 240 and 360 min) demonstrated the highest accuracy with an adjusted *r* ^2^ of 0.965. Internal validation confirmed the models’ stability and accuracy, with the four-point model exhibiting the best performance. External validation using three additional cases supported the predictive accuracy of the model.

**Conclusion:**

The LSS model developed in this study accurately predicts the Bu AUC_0-360_ with 2–4 sampling points, offering a practical and clinically valuable tool for therapeutic drug monitoring in pediatric HSCT recipients. The four-point model was found to be the most accurate and is recommended for clinical applications.

## 1 Introduction

Busulfan (Bu) is an alkylating agent used in chemotherapy regimens alongside drugs such as cyclophosphamide (CY) and fludarabine (Flu) for hematopoietic stem cell transplantation (HSCT). Owing to concerns about the long-term effects of total-body irradiation in children, Bu-based conditioning regimens have been widely applied in the conditioning of pediatric hematopoietic stem cells. However, Bu has a narrow therapeutic window, and its pharmacokinetic characteristics show significant inter-individual variability, which is particularly pronounced in children (Marsit et al., 2020). Insufficient drug exposure is associated with a higher rate of transplant failure or relapse, while excessive exposure is associated with increased toxicity and transplant-related mortality (Bartelink et al., 2016). Notably, the efficacy and adverse drug reactions of Bu are closely related to the area under the concentration-time curve (AUC) of its blood concentration, hence therapeutic drug monitoring (TDM) is often required to achieve personalized drug administration (Rasor et al., 2019; Sweiss et al., 2020; Bognàr et al., 2024). The limited sampling strategy (LSS) is a method that involves collecting a small number of samples to measure drug concentrations, using pharmacokinetic models to determine the optimal sampling times, and then assessing drug exposure and combining population pharmacokinetics to estimate the AUC. This approach minimizes blood draws, making it particularly suitable for young infants and toddlers (Sibbald et al., 2006). Although some transplant centers have established models for the LSS to predict the AUC values of Bu, the differences in patient ethnicity, age, disease types, dosing regimens, and blood sampling schemes included in different models result in poor generalizability of these models. Therefore, based on the need for clinical TDM, we aimed to develop a LSS that is more suitable for predicting Bu exposure in Chinese children undergoing HSCT at our hospital.

## 2 Materials and methods

### 2.1 Study design and population

Between October 2023 and April 2024, 26 pediatric patients receiving HSCT treatment at the Hebei Children’s Hospital were enrolled. There were 17 males and 9 females, with ages ranging from 7 months to 14 years, heights ranging from 67 to 165 cm, and weights ranging from 8.8 to 64.5 kg. The study was approved by the Ethics Committee of Children’s Hospital of Hebei Province (Approval Number: 202312), and written informed consent was obtained from the guardian of each child. The baseline data of the patients are summarized in Table 1.

**TABLE 1:** Patient characteristics.

Number of patients	*N* = 26
Age, median (range)	8.4 years (7 months–14 years)
Gender	
Male	17
Female	9
Disease	
Severe aplastic anemia	18
Acute leukemia	3
Other	5
Conditioning regimen	
Bu/CY/Flu/ATG	19
Bu/CY/Flu/TT	4
Mel/CL/Bu/CY/ATG	2
Bu/CY/TT/VP-16	1
Dose level	
1 mg/kg (<9 kg)	1
1.2 mg/kg (9–16 kg)	1
1.1 mg/kg (16–23 kg)	6
0.95 mg/kg (23–34 kg)	5
0.8 mg/kg (>34 kg)	13
Dose (mg/kg); median (range)	0.9 (0.8–1.2)

Abbreviations: Bu, Busulfan; CY, cyclophosphamide; Flu, fludarabine; ATG, antithymocyte globulin; TT, thiotepa; Mel, melphalan; CL, cladribine; VP-16, etoposide.

### 2.2 Administration and blood sample collection

All pediatric patients received a conditioning regimen based on Bu before transplantation (such as Bu/Cy/Flu + ATG, Bu/Cy/Flu/TT, etc.), with the dosage of Bu ranging from 0.8 to 1.2 mg/kg, administered as a continuous intravenous infusion over 2 h every 6 h. Patients older than 5 years were administered oral phenytoin sodium as an anticonvulsant at a dose of 5 mg/kg twice daily to prevent seizures. Epoprostenol was administered intravenously during conditioning and transplantation to prevent hepatic veno-occlusive disease. Fludarabine was administered at a dose of 30 mg/m^2^ once daily via intravenous infusion. Mesna injection, adequate hydration, and urine alkalization were performed concurrently with cyclophosphamide to prevent hemorrhagic cystitis. Cyclosporine A was administered before the infusion, and starting from day 1 post-transplantation, mycophenolate mofetil, low-dose methotrexate, and steroid hormones were used to prevent graft-versus-host disease. Venous blood was collected before and after the first dose of Bu infusion at 15, 30, 60, 120, 135, 150, 180, 240, 300, and 360 min. Blood was then centrifuged to separate the plasma, which was used for analysis.

### 2.3 Measurement of plasma Bu concentrations

Chromatography and mass spectrometric conditions: The LC-MS/MS system used for analysis included a Jasper™ HPLC system combined with a Triple Quad™ 4500MD mass spectrometer from AB SCIEX (Framingham, Massachusetts, United States). Separation of Bu in human plasma was performed using a core–shell ODS microparticulate column (Kinetex EVO C18, 2.6 µm particle size, 30 × 2.1 mm I.D.; Phenomenex, Torrance, CA, United States). The mobile phase consisted of (A) 0.07% formic acid and 2 mM NH_4_ Ac in water and (B) methanol, with the following gradient elution program: 0–0.2 min, 95% A; 0.2–0.5 min, 90%–2% A; 0.5–1.0 min, 2% A; 1.0–1.01 min, 2%–95% A; 1.01–1.8 min, 95% A. The flow rate was set at 0.5 mL/min. The column and autosampler temperatures were maintained at 45°C and 8°C, respectively. The injection volume was 1 mL. An electrospray ionization (ESI) source was used in the positive ion mode. The ESI needle voltage and nebulization temperature were kept at 4,500 V and 550°C, respectively. Curtain gas, gas 1, and gas 2 were set to 35, 60, and 60 psi, respectively. Quantification was conducted using multiple reaction monitoring with an m/z of 264.1→151.0 for Bu and 272.0→159.0 for Bu-D8 (internal standard; IS). The LC-MS/MS data were analyzed using Analyst 1.6.3 software from AB SCIEX (Framingham, Massachusetts, United States).

Sample processing: A 10 μL IS working solution was added to a 10 μL aliquot plasma sample, and 90 μL of methanol was added for protein precipitation. Then, the mixture was vortexed for 1 min and centrifuged at 12,000 × *g* for 10 min at 4°C. The supernatant from each sample was transferred to LC-MS vials for analysis.

Validation of the methodology: Validation procedures were performed according to the EMA guidelines for bioanalytical method validation (ICH, 2022). A linear relationship was observed when the concentrations of BU were between 0.025 and 16.240 μmol/L (r > 0.998). The average extract recovery for Bu was 95.83%, the matrix effects were 91.5%–99.5%, and the intra- and inter-day RSD values were less than 15%. The stability of BU was acceptable, and the established method was confirmed to be reliable for use in the pharmacokinetic studies of Bu.

### 2.4 Data statistics and analysis

#### 2.4.1 Pharmacokinetic parameters

The pharmacokinetic parameters were calculated using DAS 2.0 software (Chinese Pharmacological Society, Beijing, China) according to a non-compartment model. The peak plasma concentration (C_max_) and the time to reach peak plasma concentration (T_max_) were obtained directly from the data. Other parameters were calculated, including elimination half-life (t_1/2_), peak concentration (C_max_), AUC, clearance (CL), and apparent volume of distribution (Vd). The pharmacokinetic parameters are listed in Table 2.

**TABLE 2:** Pharmacokinetic parameters of HSCT patients after the first dose of Bu (*N* = 26).

Parameters	Units	Mean ± SD
t_1/2_	min	136.84 ± 38.60
T_max_	min	139.62 ± 9.27
C_max_	μmol/L	4.05 ± 0.61
AUC_0-t_	μmol·min/L	845.54 ± 111.03
AUC_0-∞_	μmol·min/L	1118.84 ± 199.10
MRT_0-t_	min	181.37 ± 10.55
MRT_0-∞_	min	272.43 ± 50.18
CL	L/h/kg	0.23 ± 0.04
Vd	L/kg	0.73 ± 0.15

Abbreviations: t_1/2_, elimination half-life; T_max_, time to reach peak plasma concentration; C_max_, peak plasma concentration; AUC, concentration-time curve; MRT, mean residence time; CL, clearance; Vd, apparent volume of distribution.

#### 2.4.2 Statistical analysis

Multivariate linear regression models were used to calculate Bu concentrations in the 26 patients using SPSS 25.0 (IBM SPSS, Armonk, NY, United States) software. Stepwise linear regression was performed on all possible combinations of concentrations at time points 1, 2, 3, and 4 to establish LSS models for evaluating the AUC. In addition, a stepwise forward multivariate regression method was employed to eliminate variables without statistical significance, and the regression equation with the best-adjusted coefficient of determination (*r* ^2^) was selected for model validation. An *r* ^2^ greater than 0.9 is considered the standard, indicating a good correlation between AUC_0-360_ and the predicted values. This analysis produced the following prediction formula (A was the partial correlation coefficient and C was the Bu concentration):

$$\text{Predicted }{\text{AUC}}_{0-360}=\text{Intercept}+A_{1}\times C_{1}+A_{2}\times C_{2}+\ldots +A_{n}\times C_{n}$$

The prediction error (PE), absolute prediction error (APE), and root-mean-square error (RMSE) were calculated using the following equations to evaluate predictive bias, accuracy, and precision:

$$PE\%=\frac{\left(predictedAUC-actualAUC\right)}{actualAUC}\times 100\%$$

$$APE\%=\frac{\left|predictedAUC-\left.actualAUC\right|\right.}{actualAUC}\times 100\%$$

$$RMSE\%=\sqrt{1/n\sum {\left(PE\%\right)}^{2}}$$

#### 2.4.3 Model validation

Two different methods were employed for internal validation of the model. The Jackknife method was used for the internal validation of the model. In each iteration, one sample was removed from the original sample set (*n* = 26) and a regression analysis was conducted. The pharmacokinetic parameters of the excluded samples were calculated using the derived regression equation. The accuracy and precision of the regression equation were evaluated using the PE and RMSE. The closer the PE% is to 0 and the smaller the RMSE, the fewer the number of samples with prediction errors in Bu parameters exceeding ±15%, indicating better accuracy and precision for the regression equation. The optimal models for one, two, three, and four sampling points were subjected to the bootstrap method, with 2,000 resampling iterations to calculate the median and 95% confidence interval (CI). The procedure was performed using R software (version 3.0).

#### 2.4.4 Method consistency evaluation

The consistency between the measured values of Bu and predicted values of Bu was assessed using the intraclass correlation coefficient (ICC) and Bland–Altman (BA) analysis method. An ICC with a lower limit of the 95% CI exceeding 0.9 is considered a good indicator of consistency between the classical method and LSS method. In the BA analysis graph, 95% of the points should be within the limits of agreement, and these limits of agreement should not exceed the professionally acceptable critical value range (15%). Generally, if these two conditions are satisfied, the consistency between the two methods is considered good.

#### 2.4.5 External validation

In addition, we used three pediatric cases that were not included in the linear regression model to perform a small-sample external validation of the established model. For these three patients, AUC calculations were conducted using both the traditional pharmacokinetic method and LSS methods established in this study, and the PE was calculated.

## 3 Results

### 3.1 Concentration–time curves

**FIGURE 1:**
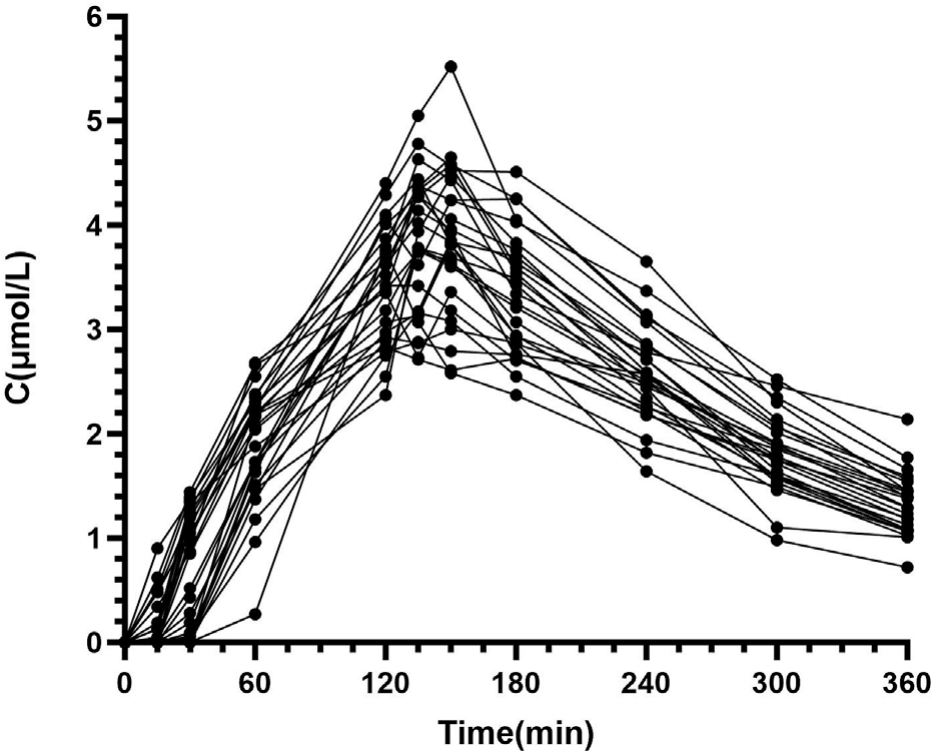
Individual blood concentration-time curves of BU in patients undergoing HSCT (*N* = 26).

**FIGURE 2:**
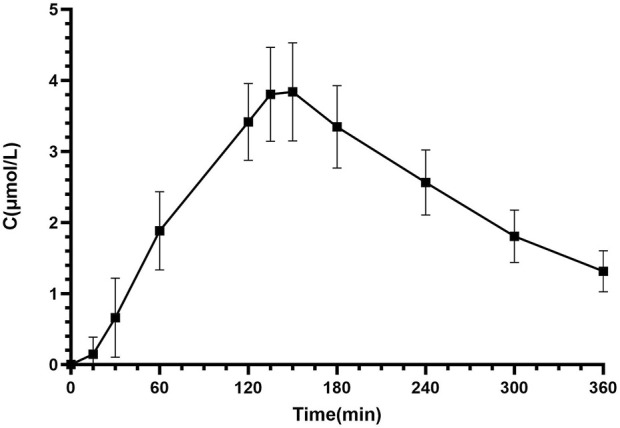
Mean AUC of intravenous Bu in patients undergoing HSCT (*N* = 26).

Pharmacokinetics samples (total: 286 concentrations) were collected from all 26 patients. The blood concentration (μmol/L) versus time (min) curves for the first intravenous administration of Bu are depicted in Figures 1, 2.

### 3.2 Pharmacokinetics

The mean ± standard deviation (SD) for AUC_0-t_, mean residence time (MRT)_0-t_, CL, and Vd of these 26 patients were 845.54 ± 111.03 μmol min/L, 181.37 ± 10.55 min, 0.23 ± 0.04 L/h/kg, and 0.73 ± 0.15 L/kg, respectively. The other non-compartmental statistical moment parameters are presented in Table 2.

### 3.3 LSS predicted AUC_0-360_

**FIGURE 3:**
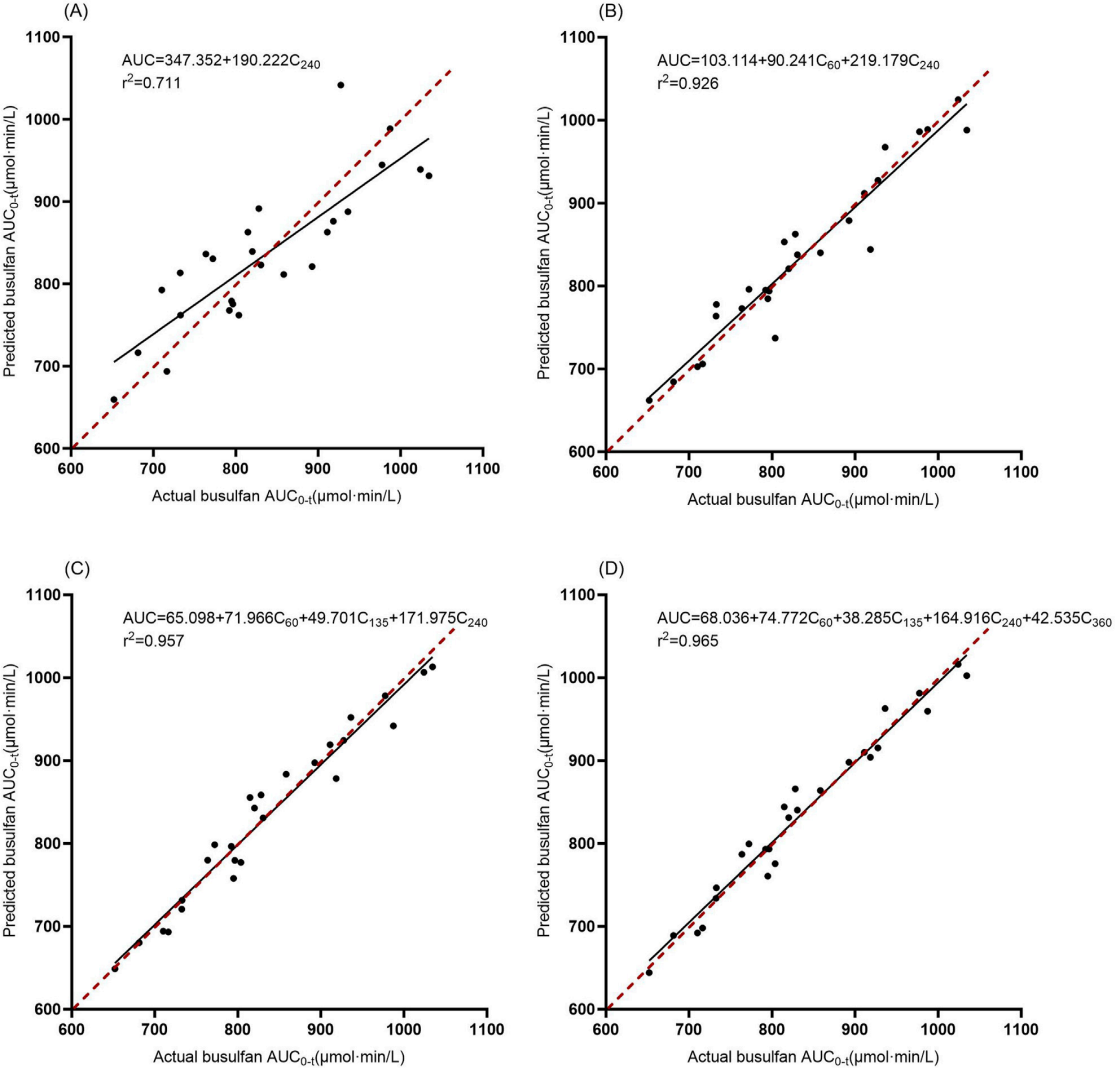
Correlation between the actual and estimated BU AUC_0-360_ in models involving 1–4 time points. The solid line is the regression line from the data, and the dashed line is the ideal prediction line where predictions equal actual values. **(A)**: Model 1, **(B)**: Model 4, **(C)**: Model 7, **(D)**: Model 10.

After evaluating all combinations for use in estimating AUC_0-360_ from 1–4 blood sampling points, the top three equations with the largest adjusted coefficients of determination for each were selected, totaling four equations, and the results are shown in Table 3. The accuracies of the different regression equations are compared in Table 4 and Figure 3. The results showed that in the regression equations predicting the AUC_0-360_ from a single blood concentration–time point, the adjusted *r* ^2^ was less than 0.9, indicating a weak correlation. In contrast, the regression equations predicting AUC_0-360_ from two, three, and four sampling points exhibited a better linear relationship. Among them, the equation using multivariate regression of AUC_0-360_ against C_60_, C_135_, C_240_, and C_360_ exhibited the best linear relationship and prediction effect. The equation using C_60_ and C_240_ for multivariate regression also met the prediction requirements.

**TABLE 3:** Multiple linear regression models of Bu after the first dose (*N* = 26).

Model No.	Sampling time (min)	Regression equation	*r* ^2^
1 time point model			
1	240	347.352 + 190.222C_240_	0.711
2	150	349.904 + 125.223C_150_	0.671
3	135	352.580 + 125.256C_135_	0.620
2 time point models			
4	60, 240	103.114 + 90.241C_60_ + 219.179C_240_	0.926
5	120, 240	149.983 + 77.676C_120_ + 162.012C_240_	0.821
6	135, 240	217.361 + 75.016C_135_ + 127.897C_240_	0.827
3 time point models			
7	60, 135, 240	65.098 + 71.966C_60_ + 49.701C_135_ + 171.975C_240_	0.957
8	60, 150, 240	70.648 + 79.648C_60_ + 53.081C_150_ + 158.367C_240_	0.940
9	60, 120, 240	65.098 + 71.630C_60_ + 48.166C_120_ + 195.662C_240_	0.930
4 time point models			
10	60, 135, 240, 360	68.036 + 74.772C_60_ + 38.285C_135_ + 164.916C_240_ + 42.535C_360_	0.965
11	60, 150, 240, 360	66.552 + 79.381C_60_ + 45.042C_150_ + 142.387C_240_ + 59.785C_360_	0.961
12	60, 120, 240, 360	31.474 + 72.184C_60_ + 41.931C_120_ + 174.841C_240_ + 56.712C_360_	0.953

**TABLE 4:** Comparison of the accuracy of different regression equations (*N* = 26).

Model No.	Sampling time (min)	PE		APE		10%^a^	15%^b^
		Range (%)	Mean ± SD	Range (%)	Mean ± SD		
1	240	−12.30–9.96	−0.43 ± 6.63	0.12–12.30	5.59 ± 3.42	3	0
4	60, 240	−6.11–8.30	−0.11 ± 3.39	0.01–8.30	2.31 ± 2.44	0	0
7	60, 135, 240	−5.01–4.66	0.28 ± 2.65	0.05–5.01	2.11 ± 1.58	0	0
10	60, 135, 240, 360	−4.59–4.30	−0.04 ± 2.37	0.12–4.59	1.89 ± 1.37	0	0

Note: PE: prediction error; APE: absolute prediction error.

^a^ The number of APEs, exceeding 10%.

^b^ The number of APEs, exceeding 15%.

### 3.4 Validation of the LSS model

The adjusted coefficient of determination of the multiple regression equation can serve as an important indicator of the quality of the model; however, it does not reflect its stability. Therefore, the Jackknife method was used for internal validation. The results of the Jackknife validation are listed in Table 5. When modeling AUC_0-360_ with only one parameter, one prediction result had an error exceeding 15%, while four prediction results had errors between 10% and 15%. However, when predicting with two or more parameters, the PE did not exceed 95%. The RMSE also decreased with an increase in the number of sampling points. At the same time, the 95% confidence intervals for the model parameters of the four best combinations were calculated using the bootstrap method. The 95% confidence intervals were obtained using 2,000 systematic bootstrap samples, and the results are shown in Table 6.

**TABLE 5:** Validation of LSS models using Jackknife method (*N* = 26).

Sampling time (min)	PE% (mean ± SD)	APE% (mean ± SD)	RMSE	10%^a^	15%^b^
240	0.55 ± 7.31	6.08 ± 3.91	7.06	4	1
60, 240	−0.49 ± 3.67	2.56 ± 2.62	3.56	0	0
60, 135, 240	−0.44 ± 3.08	2.46 ± 1.83	2.99	0	0
60, 135, 240, 360	−0.45 ± 2.97	2.42 ± 1.71	2.89	0	0

Note: PE, prediction error; APE, absolute prediction error.

^a^ The number of APEs, exceeding 10%.

^b^ The number of APEs, exceeding 15%.

**TABLE 6:** Results of different LSS models calculated using the Bootstrap method.

Model No.	Intercept^a^	M_1_ ^b^	M_2_	M_3_	M_4_
1	347.352 (182.235–465.817)	190.222 (141.453–255.353)			
4	103.114 (27.538–182.879)	90.241 (70.219–108.893)	219.179 (196.303–241.801)		
7	75.589 (22.716–135.825)	76.093 (58.389–96.683)	38.443 (14.975–62.042)	182.728 (145.604–209.473)	
10	68.036 (3.341–132.992)	74.772 (60.109–100.202)	38.285 (12.435–58.039)	164.916 (124.047–217.061)	42.535 (−33.177–76.429)

^a^ Median (95% CI) of the intercept.

^b^ Median (95% CI) of the coefficient (M_1-4_).

### 3.5 Evaluation of the LSS model

**FIGURE 4:**
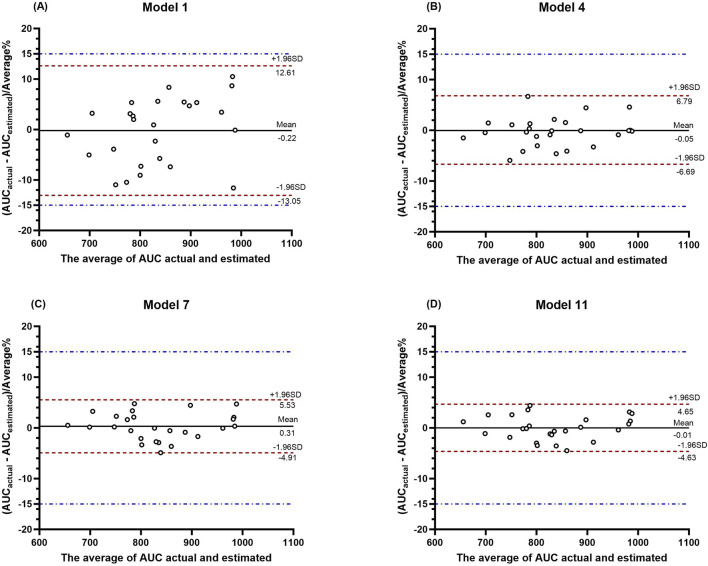
BA plot analysis of the actual AUC values versus the predicted AUC values from LSS. **(A–D)** show the BA plot analysis for the four models, respectively.

The ICC and BA analysis results are presented in Table 7. It can be observed that the two-point, three-point, and four-point sampling equations yielded AUC predictions with ICC values, where the lower limits of the 95% confidence intervals all exceeded 0.9 when compared to the AUC values obtained by the classical method. A graphical analysis of the BA for the LSS is shown in Figure 4, where all points fall within the 15% range. As the number of sampling points increased, the mean error approached the zero-error line, and all points became more concentrated near the zero-error line. These results demonstrate that two or more sampling points provide good consistency with the classical method, with the four-point method showing the best performance.

**TABLE 7:** Consistency evaluation results between the LSS and classical methods.

Model No.	Sampling time (min)	ICC	95% CI
1	240	0.837	0.668–0.923
4	60, 240	0.963	0.920–0.983
7	60, 135, 240	0.978	0.951–0.990
10	60, 135, 240, 360	0.983	0.962–0.992

Note: ICC, intraclass correlation coefficient.

### 3.6 External validation

From the external validation results of the three patients, model 1 had poor reliability, with a PE% exceeding 15%. Although model 4 had a PE% within 10%, its stability was not satisfactory. Models 7 and 10 generally provided predictions closer to the actual AUC_0-360_ values, with model 10 showing the best predictive performance for all three patients and exhibiting the best stability. See Table 8 for more details.

**TABLE 8:** External validation results for the best linear regression models from 1 to 4 time points.

Patient No.	Actual AUC_0-360_	Predicted AUC_0-360_				PE%			
		Model 1	Model 4	Model 7	Model 10	Model 1	Model 4	Model 7	Model 10
1	1024.05	938.94	1024.80	1006.58	1016.20	−8.31	0.07	−1.71	−0.77
2	717.975	836.22	787.33	746.65	737.84	16.47	9.66	3.99	2.77
3	1160.02	961.77	1149.47	1137.96	1164.92	−17.09	−0.91	−1.90	0.42

Note: PE, Prediction error.

## 4 Discussion

The efficacy and adverse drug reactions of Bu are closely related to the AUC. As a cytotoxic drug, Bu has a narrow therapeutic window and significant inter-individual pharmacokinetic variability, particularly in children. Therefore, TDM is often required to achieve personalized dosing. The pharmacokinetic data from this study align with those from a previous study (Cremers et al., 2002) for the first dose administration (population estimates: CL = 0.29 L/h/kg and Vd = 0.84 L/kg). However, it has been reported that there is significant intra-individual variability in the CL of Bu when used in children prior to HSCT, with approximately 80% of patients experiencing a decrease in CL (Marsit et al., 2020). Since our study only included CL values from the first Bu dose, these did not represent the CL at steady state. Some studies have suggested that age is the primary factor affecting Bu exposure in children (Cremers et al., 2002; Faraci et al., 2018; Nakamura et al., 2008). Other studies have demonstrated a close logarithmic linear relationship between body weight and Bu CL, suggesting that weight-based dosing might be more appropriate for pediatric patients (Nguyen et al., 2004). Unlike other published studies, the predominant disease in our study was severe aplastic anemia, a non-malignant condition, and the proportion of younger children was relatively low, which may have led to certain differences in the pharmacokinetic data compared to other studies.

Although some studies have successfully established models to estimate the AUC_0-t_ values of Bu using the LSS method during the pre-experimental phase, our attempts to validate some of these established models yielded unsatisfactory results, with significant percentage errors (PE). As is well known, the classic pharmacokinetic calculation of AUC is performed by summing up trapezoidal areas, and a greater number of blood sampling points can yield a more accurate AUC value. Therefore, we collected 11 blood samples per patient, which allowed a more precise reflection of drug exposure in children, thereby facilitating external validation and model generalization. In pediatric patients, Bu is administered as a continuous 2-h intravenous infusion, and the longer infusion tubing leads to a delay in the time to reach peak concentration (Neroutsos et al., 2021). We studied and compared the blood sampling schemes of other LSS models. To better capture the C_max_ value, we designated three blood sampling time points at 120, 135, and 150 min post-administration. The results of our study indicate that the majority of patients had the highest blood concentration 135 min after administration. In addition, significant fluctuations in blood concentrations around C_max_ were evident. If peak concentration is only measured at two time points (120 and 150 min), the maximum peak concentration may be underestimated. In the present study, blood samples for Bu were collected from a separate venous fluid pathway to limit the influence of drugs on the fluid pathway. Therefore, the design of the blood sampling scheme is particularly important for accurately calculating the AUC (Utano et al., 2021; Teitelbaum et al., 2020; Watanabe et al., 2015). Multiple linear regression (MLR) is a statistical method of regression analysis used to analyze and model the linear relationship between a dependent variable and multiple independent variables. This approach helps assess the effect of each independent variable on the dependent variable and allows for predictions. The application of MLR models to predict drug AUC is becoming increasingly popular (Li et al., 2022; Xiang et al., 2021).

Theoretically, drug concentrations may fluctuate during the infusion period due to dynamic changes in absorption and distribution. After the infusion is complete, drug concentrations may stabilize or begin to decline. The calculation of CL is based on the elimination phase of the drug, which can more accurately reflect the drug’s clearance characteristics. However, our established MLR model showed that the best-adjusted *r* ^2^ values in the equations included the independent variable C_60_, suggesting that the blood concentration halfway through infusion may have some predictive value for subsequent changes in blood concentration. Therefore, our data processing experience suggests that including samples collected during the infusion period may enhance the predictive accuracy of MLR equations.

The Jackknife method, also known as “leave-one-out cross-validation,” assesses the stability of a statistic on data subsets by repeatedly excluding each observation and recalculating the statistic. This is useful for robustness analysis of parameter estimations in MLR models. Bootstrapping is a nonparametric method that estimates the sampling distribution of a statistic by resampling with replacements from the original data. This method allows for a more accurate estimation of model performance indicators (such as regression coefficients) because it considers variability in the sample data. Using both the Jackknife and Bootstrap methods for the internal validation of MLR models can provide a more comprehensive, robust, and precise model assessment, especially when dealing with small samples, non-normally distributed data, and complex data characteristics. The combined use of these methods can enhance the reliability and effectiveness of model validation. The ICC assesses consistency by calculating correlation coefficients, which are suitable for evaluating the consistency of multiple raters or multiple measurements, while BA plots are useful in assessing consistency by comparing the average differences and limits of agreement between two measurement methods, suitable for evaluating the consistency between two different measurement techniques or raters. In consistency evaluation, combining both methods for joint assessment can effectively avoid the limitations of a single-method evaluation, providing more representative results.

In this study, we conducted an external validation of the model using three patients and found good consistency with the internal validation results, which also met the clinical requirements for AUC accuracy. Subsequently, all children undergoing HSCT with Bu received AUC_0-360_ predictions using model 10 with four blood sampling points, which reduced manpower and patient costs while providing effective personalized medication data. Nevertheless, our study has certain limitations. Because we used a more intensive blood sampling scheme that included times covering the absorption, distribution, metabolism, and elimination phases of the drug, the number of children enrolled in the study, sample size for external validation, and the proportion of infants and young children were all relatively small. Moreover, our data collection was limited to a specific region, which may restrict the generalizability of our results. Additionally, owing to funding and time constraints, we did not conduct long-term follow-up studies to provide clinical outcomes for children based on different AUC levels of Bu. We will address these issues in subsequent research.

In conclusion, this study employed MLR to establish a LSS model, and the results indicated that when the number of sampling points is 2–4, it can accurately predict the AUC_0-360_ values of Bu. The model was internally validated using both the Jackknife and Bootstrap methods, and methodological consistency was evaluated using the ICC and BA methods. When the number of sampling points was 2–4, the accuracy and stability of the model improved with an increase in the number of sampling points. From the external validation data, it was observed that the predictive performance was better when the number of sampling points was 3–4; thus, it is recommended to use either three or four blood sampling points for prediction in practical applications. In summary, the LSS model, as a mathematical-pharmacological method with strong operability, demonstrates good clinical application value for TDM of Bu in patients undergoing HSCT.

## Data Availability

The original contributions presented in the study are included in the article/supplementary material, further inquiries can be directed to the corresponding author.
